# Electroacupuncture improves scopolamine hydrobromide induced dry eye in mice via inhibiting ocular surface inflammation and regulating the HMGB1-related signaling pathways

**DOI:** 10.3389/fmed.2025.1664376

**Published:** 2025-10-23

**Authors:** Xia Wu, Ning Ding, Shangjie Liang, Yutong Han, Siyuan Fan, Yunchuan Wu, Qingbo Wei

**Affiliations:** School of Acupuncture-Moxibustion and Tuina, School of Health Preservation and Rehabilitation, Nanjing University of Chinese Medicine, Nanjing, Jiangsu, China

**Keywords:** dry eye disease, electroacupuncture, high mobility group protein B1, receptor for advanced glycation end products, ocular surface inflammation

## Abstract

**Objective:**

This study aims to observe the effect of Electroacupuncture (EA) on improving ocular surface inflammation and *HMGB1*-related signaling pathways in dry eye disease (DED). Methods: Healthy male C57BL/6 J mice were treated with scopolamine hydrobromide for 21 consecutive days to establish the animal models for DED. After 21 days, fluorometholone (Flu), EA, and sham EA (Sham) treatments were performed. The effect of EA on DED surface inflammation was evaluated by corneal fluorescence staining, phenol red thread test, *in vivo* confocal microscopy (IVCM), and corneal histopathology. The influence of EA on high-mobility group box 1 (*HMGB1*), receptor for advanced glycation end products (*RAGE*), toll-like receptor 2 (*TLR2*) and toll-like receptor 4 (*TLR4*) was assessed by immunohistochemistry, real-time quantitative polymerase chain reaction (RT-qPCR), and western blot. The influence of EA on interleukin-6 (IL-6) and interleukin-10 (IL-10) were measured by enzyme-linked immunosorbent assay (ELISA).

**Results:**

EA can significantly increase tear flow and reduce corneal staining and corneal stromal inflammation, while also improving the morphologic structure of the cornea and lacrimal glands. The levels of HMGB1, RAGE, TLR2, TLR4, and IL-6 were significantly decreased while IL-10 level was significantly increased after EA treatment, indicating that EA may improve dry eye surface inflammation by inhibiting HMGB1-related signaling pathways.

**Conclusion:**

The findings presented in our study demonstrate that EA may improve ocular surface inflammation in mice with DED by inhibiting the HMGB1-related signaling pathways. Therefore, EA may be a potential therapeutic target for DED.

## 1 Introduction

DED is the most common multifactorial chronic ocular surface disease in clinical practice, with a prevalence of 5 to 50% (1, 2), which not only seriously affects the quality of life of patients (3), but also causes a huge social and personal economic burden (4). The latest TFOS DEWS III report indicates that DED is characterized by a loss of homeostasis of the tear film and/or ocular surface, in which tear film instability and hyperosmolarity, ocular surface inflammation and damage, and neurosensory abnormalities are etiological factors (5). Dryness, foreign body sensation, light sensitivity, itching, and blurred vision are common symptoms of DED. DED can cause damage to the corneal epithelium, and in severe cases, can progress to secondary keratitis, corneal ulcers, scleritis, uveitis, optic neuritis, and irreversible vision loss (6). The chronic inflammatory response of the ocular surface is one of the recognized core mechanisms of DED (7). At present, the first-line treatment of DED focuses on fully relieving symptoms. Fluorometholone, a synthetic glucocorticoid, is widely prescribed for the management of DED. Fluorometholone can inhibit ocular surface inflammation, reduce the further damage of lacrimal gland and meibomian gland, and prevent DED from developing into corneal ulcer or irreversible optic nerve damage (8). Nevertheless, the curative effect of fluorometholone is limited. It is important to note that the discontinuation of fluorometholone can result in the reappearance of DED. It is well-documented that the long-term use of fluorometholone can result in elevated intraocular pressure, the development of a secondary infection, and a range of other adverse effects (9).

**Figure 1:**
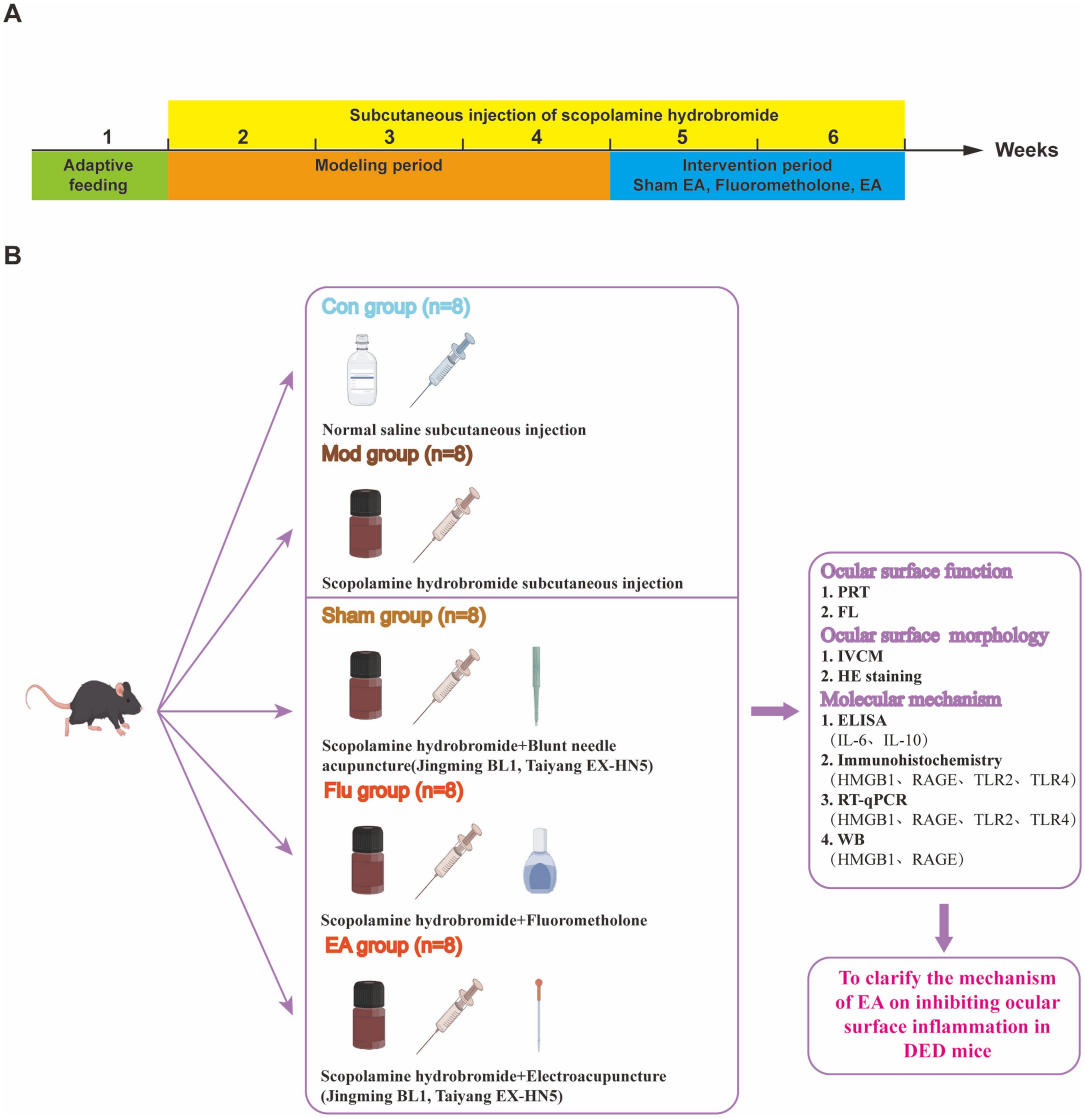
**(A)** Different operations on mice at different time points; **(B)** Experimental strategies and main indicators in mice.

Acupuncture regulating the physiological state of the human body through stimulation of specific parts of the body (acupuncture points), is one of the most common complementary and alternative therapies available for treating diseases (10). EA has been widely used in both fundamental research and clinical practice as a therapeutic modality that integrates electrophysiological techniques and acupuncture. Previous studies have shown that EA treatment for DED is both efficacious and safe (11–14), which can improve ocular surface inflammation and promote tear secretion in DED animal models (15–19). So far, the specific mechanism of EA in treating DED has not been fully elucidated. Current research suggests that the effects of EA are multitargeted, encompassing the amelioration of structural and functional ocular surface abnormalities, modulating apoptosis and autophagy in the cells of the ocular surface, stimulating neurotransmitter secretion, and suppressing immunologic inflammatory responses (12). A recent study suggests that damage-associated molecular pattern (DAMP) is now considered a key regulatory factor for innate and adaptive immunity through receptor-mediated signaling mechanisms (20). DAMPs activate downstream signaling cascades by binding to receptors, regulating key biological processes including cell proliferation, differentiation, migration, and inflammation (21). Furthermore, *HMGB1*, as one of the important molecules in DAMP, has been described as a key cytokine involved in cell activation and proinflammation in recent years (22, 23). On the one hand, the negative regulatory effect of *HMGB1* on regulatory T cells makes it play an important role in adaptive immunity (24, 25). On the other hand, *HMGB1* released into the extracellular space mediates the activation of innate immune responses. It binds to various extracellular receptors (*RAGE*, *TLR2*, *TLR4* and others) and activates the release of inflammatory cytokines from macrophages and dendritic cells, causing the rupture of macrophage lysosomes and the release of calcium antagonist peptides into the cytoplasm, and further triggering immune and inflammatory responses in the body and exacerbating tissue damage (26, 27). Recent experimental studies have demonstrated that EA can inhibit the expression of *HMGB1* (28, 29). However, the specific regulatory mechanism of EA inhibiting *HMGB1* to reduce the ocular surface inflammation of DED is not fully understood. Therefore, the aim of this study was to elucidate the mechanism by which EA inhibits DED surface inflammation through the *HMGB1*-related signaling pathway by assessing the expression levels of *HMGB1* and its receptors in DED mice tissues (Figure 1), and to provide a basis for the beneficial role of EA in the pathogenesis of DED.

## 2 Materials and methods

### 2.1 Experimental animals

Forty specific pathogen free-grade male C57BL/6 J mice, weighing 18-20 g, were provided by Beijing Vital River Laboratory Animal Technology Co., Ltd., and reared in the Animal Experiment Center of Nanjing University of Chinese Medicine (Animal Qualification Certificate No: 110011231108729257). Before the experiment began, all mice were given adaptive feeding for one week. Feeding environment: room temperature 20–26 °C, relative humidity 40–70%, 12 h light/dark cycles and free diet. Only mice with normal eye surface function were used for the experiment. All animal experiments were conducted in accordance with the guiding principles and ethical standards of the Animal Ethics Committee of Nanjing University of Chinese Medicine (Ethics No. 202306A035).

### 2.2 Instruments and reagents

Instruments and reagents used in this study: LYL-S handheld slit lamp microscope inspection instrument (Shangrao Rixin Optical Instrument Components Co., Ltd., China); acupuncture needle (φ0.18 × 13 mm, Huatuo brand, Suzhou Medical Supplies Factory Co., Ltd., China); SDZ-II electroacupuncture therapy device (Huatuo brand, Suzhou Medical Supplies Factory Co., Ltd., China); Confocal Microscopy (HRT III RCM, Heidelberg Company, Germany); RM2016 pathological slicer (LEICA, Germany); optical microscope (Nikon, Japan); fluorometholone eye drops (0.1%, Shentian Pharma, China); scopolamine hydrobromide (Chengdu Pufeide Biotech Co., Ltd., China); tear detection phenol red thread (Tianjin Jingming New Technology Development Co., Ltd., China); fluorescein sodium ophthalmic strips (Tianjin Yino Xinkang Medical Equipment Technology Co., Ltd., China); IL-6 ELISA Kit (BMS603-2HS, Invitrogen, America); IL-10 ELISA Kit (CSB-E04594m-IS, CUSABIO, China); SuperMix for qPCR (gDNA digester plus) (Yisheng Biotechnology Co., Ltd., China); qPCR SYBR Green Master Mix (Yisheng Biotechnology Co., Ltd., China); HRP-conjugated goat anti-rabbit IgG (GB23303, Servicebio, China); HMGB1 (GB11103-100, Servicebio, China); RAGE (PA1-075, Invitrogen, America); TLR2 (PA1-41045, Invitrogen, America); TLR4 (MA5-16216, Invitrogen, America).

### 2.3 Experimental design

Forty healthy male C57BL/6 J mice were randomly divided into control (Con), model (Mod), sham EA (Sham), fluorometholone (Flu), and EA groups, with 8 mice in each group. The experiment was designed for a total of 35 days, of which the DED mouse models were established for the first 21 days and the treatments were carried out for 14 days starting from the 22nd day. We established mouse models of DED induced by scopolamine hydrobromide, following our previous study (18). In the first 21 days, the Con group received subcutaneous injection of 200 μ L of physiological saline, while the other 4 groups received subcutaneous injection of 200 μL of scopolamine hydrobromide (0.5 mg of scopolamine hydrobromide dissolved in 0.2 mL of sterile physiological saline) to establish DED models, 4 times a day (8:00, 11:00, 14:00, 18:00). Treatment was started after completing the DED model on the 22nd day. The Con and Mod groups were treated as in the first 21 days. Sham group: sham EA treatment (Jingming BL1, Taiyang EX-HN5), which is blunt needle acupuncture without skin penetration, was administered once daily for 14 consecutive days. Flu group: fluorometholone eye drops were performed to both eyes 3 times a day (8:00, 13:00, and 18:00) for 14 days. EA group: EA treatment was performed on the same acupoints as the Sham group, with sparse wave, frequency of 2 Hz/20 Hz and current intensity of 1 mA, retaining the needle for 15 min, once a day, for 14 days. The Sham, Flu, and EA groups continued to receive subcutaneous injections of scopolamine hydrobromide during treatment to maintain the DED model. At 1, 7, 14, 21, 28, and 35 days, tear production (Phenol Red Thread Test, PRT) and fluorescein staining score (FL) were measured. Moreover, confocal microscope examination was performed on day 35. All mice were then euthanized by rapid cervical dislocation after being anesthetized. According to the double-blind principle, without knowing the grouping, data collection was conducted by one person and analysis was conducted by another person throughout the experiment.

### 2.4 PRT

The end of a phenol red thread was put into the conjunctival sac outside one third of the lower eyelid of the mouse. After 20 s, the phenol red thread was taken out for measuring the length of red-colored thread. The tear volume was determined by the length of red-colored thread.

### 2.5 Corneal fluorescein staining score

Strips of corneal staining filter paper were moistened with physiological saline and placed into the fornix of the lower eyelid of the mouse. Mice distributed fluorescein quickly and evenly across the cornea by blinking. The epithelial corneal injury was evaluated by slit-lamp examination of corneal epithelium stained with fluorescein sodium and grading with cobalt blue. According to the corneal lesion classification method, the cornea was divided into four quadrants (30). The scoring criteria for each quadrant were as follows: no staining is worth 0 points, less than 5 stains is worth 1 point, 5 or more stains is worth 2 points, and the presence of patchy staining or filaments is worth 3 points. The total score for a single eye was the sum of scores from four quadrants, with a maximum possible score of 12 points.

### 2.6 *In vivo* confocal microscopy

Mice were surface anesthetized with 0.4% obucaine hydrochloride eye drops after the head was fixed under a confocal corneal microscope. After the eyelid fixator was secured, Vidisci clear gel was applied to the surface of the conical objective lens with a 40x water immersion objective, which was then slowly advanced. When the corneal endothelial cells were clearly centered in the display, the record button was pressed to save the images of each layer of the cornea.

### 2.7 Hematoxylin and eosin staining

A portion of the corneal and lacrimal gland tissues was excised, immersed in 4% paraformaldehyde, and fixed for 24 h after euthanizing the mice on day 35 of the experiment. Then, paraffin embedding, sectioning, gradient ethanol hydration, HE staining, dehydration and sealing were performed. After staining, the morphological changes in mouse cornea and lacrimal gland were observed under an upright optical microscope.

### 2.8 Enzyme-linked immunosorbent assay

The whole blood of mice was placed at room temperature for 2 h followed by centrifugation at 3,000 rpm for 10 min at 4 °C, and the supernatant was collected for analysis. The levels of IL-6 and IL-10 were determined by enzyme-linked immunosorbent assay (ELISA) kits.

### 2.9 Immunohistochemistry

Four percentage paraformaldehyde was used to fix mouse cornea and lacrimal gland tissues. Antigen repair was performed with EDTA antigen repair solution [pH = 9.0 after paraffin sectioning, dewaxing, and hydration. 3% bovine serum albuminutes (BSA) was used to close for 30 min at room temperature]. Primary antibody HMGB1 (1:300), RAGE (1:50), TLR2 (1:100), and TLR4 (1:100) were added dropwise. After incubated at 4 °C overnight, the sections were reacted with HRP-conjugated secondary antibody (1:200) at room temperature for 50 min. Diaminutesobenzidine (DAB) solution was used to develop the staining. The positive expression rate was analyzed and calculated using Image-Pro Plus 6.0 software.

### 2.10 Reverse transcription real-time quantitative polymerase chain reaction

Trizol method was used to extract total RNA from mice lacrimal glands, which was then converted to *cDNA* with a reverse transcription kit. RT-PCR was performed in a 10 μL reaction system containing *cDNA* SYBR Green Master Mix and specific primers according to the manufacturer’s instructions, with the following specific reaction process: pre-denaturation at 95 °C for 5 min, denaturation at 95 °C for 10 s, and annealing at 60 °C for 30 s in 40 consecutive cycles. The primers were designed for *GAPDH*, *HMGB1*, *RAGE*, *TLR2* and *TLR4*, with *GAPDH* used as the internal reference. The sequencing information for each primer pair is shown in Table 1. Finally, the 2^−ΔΔCt^ method was used to determine the relative expression level of the *mRNAs*.

**Table 1:** Primer sequences for RT-qPCR.

Genes	Forward primer (5′-3′)	Reverse primer (5′-3′)	Product length
HMGB1	TGGCAAAGCAAGGAGTG	AATGGCGGTTAAAGGAGAG	135 bp
RAGE	TGGAGAGCCACTTGTGCTAA	CCCTCATCGACAATTCCAGT	191 bp
TLR2	TGCTCCTGCGAACTCCTATC	CAGACTCCAGACACCAGTGC	175 bp
TLR4	AAACGGCAACTTGGACCTG	TACTTCCTTCTGCCCGGTAA	164 bp
GAPDH	CACCCCATTTGATGTTAGTG	CCATTTGCAGTGGCAAAG	220 bp

### 2.11 Western blot

The mouse lacrimal gland tissue was grounded and treated with Radio-Immunoprecipitation buffer (RIPA) containing protease inhibitor, and lysed for 20 min on ice. The supernatant was collected by centrifugation at 12,000 rpm for 10 min at 4 °C. The concentration of the protein in the supernatant was determined according to the Bicinchoninic Acid (BCA) Protein Quantification Kit instructions and adjusted to 2 μg/μL by adding new lysis buffer. A total of 15 μL of 30 *μg* of each sample of protein was sampled for SDS-PAGE (10%) electrophoresis. The proteins were then transferred to polyvinylidene fluoride (PVDF) membranes. Following closing with quick blocking buffer for 15 min at room temperature, the membranes were immersed in 5% bovine serum albumin (BSA) containing primary antibodies against HMGB1 (1:500), RAGE (1:500) or GAPDH (1:1000), for overnight incubated at 4 °C. Next, the membranes were incubated with goat anti-rabbit IgG antibody (diluted with 5%BSA 1:15000) for 1 h at room temperature after washing 6 times with Tris-buffered saline and Tween 20 (TBST) for 5 min each. After washing again, the membrane was soaked in the super enhanced chemiluminescence (ECL) detection reagent and then placed in a chemiluminescence imager for development. For difference analysis, each group of proteins was quantified by WB using ImageJ software.

### 2.12 Statistical analyses

Data analysis was performed using the statistical software SPSS 26.0. The measurement data was expressed as mean ± standard deviation (*x ± s*) and followed a normal distribution and homogeneity of variance. One-way ANOVA followed by multiple pairwise comparisons with the Bonferroni correction was used to analyze the differences between multiple groups. Differences between two groups were compared using LSD-t test. Statistical significance was defined as *p* < 0.05.

## 3 Results

### 3.1 EA treatment increases tear flow in DED mice

**Figure 2:**
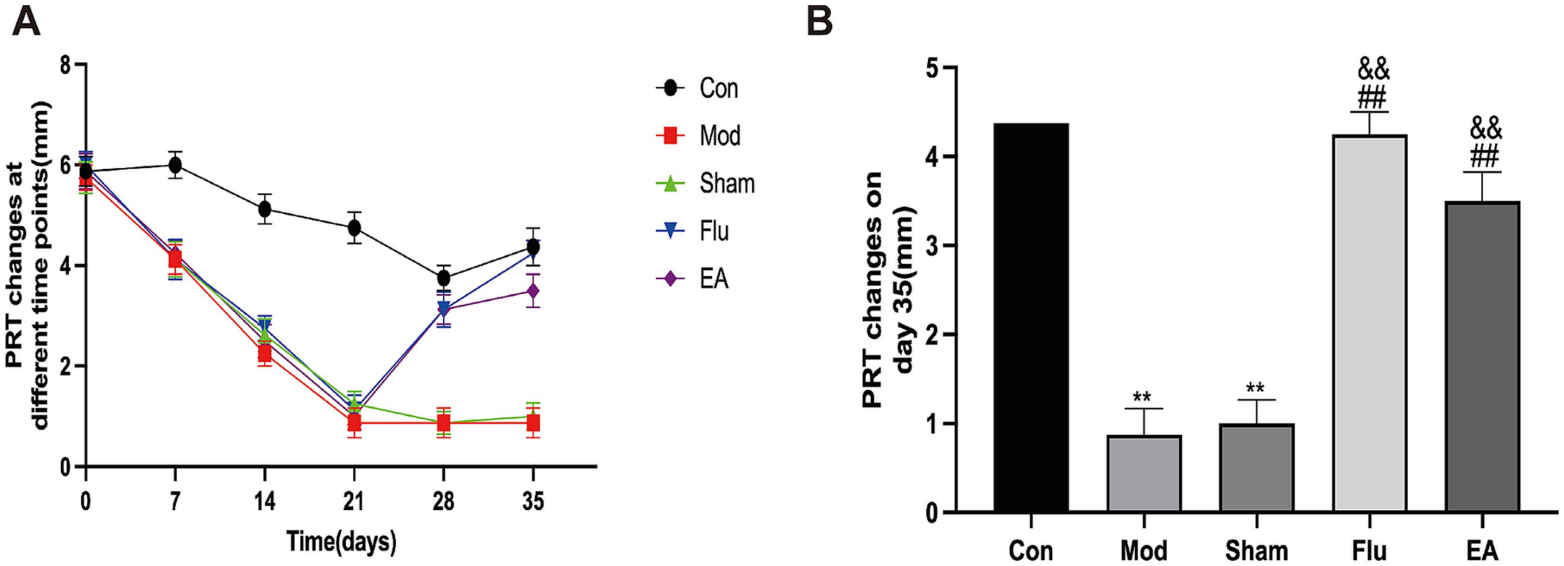
Effect of EA on tear volume in scopolamine-hydrobromide-induced DED mice. **(A)** Tear volume changes at different times (*n* = 8); **(B)** Tear volume of each of the groups on the 35th day (*n* = 8). Compared with the Con group, ^∗∗^*p* < 0.01; compared with the Mod group, ^##^*p* < 0.01; compared with the Sham group, ^&&^*p* < 0.01.

The PRT results showed that, compared with the same group before the experiment, the tear volume of the Mod, Sham, Flu and EA groups was significantly reduced on day 21 (all *p* < 0.01), and the tear volume of the Mod group on days 28 and 35 was also reduced compared with before the experiment (both *p* < 0.01), proving that the modeling was effective (Figure 2A). Tear volume was significantly lower in the Mod and Sham groups compared to the Con group and continued to decline from day 14 (Figure 2A). Tear volume was significantly increased in the EA group compared to the Mod and Sham groups after 14 days of treatment (Day 35:4.38 ± 1.06 vs. 0.88 ± 0.84 mm, *p* < 0.01; 4.38 ± 1.06 vs. 1.00 ± 0.76 mm, *p* < 0.01, Figure 2B). Tear volume was also significantly increased in the Flu group compared to the Mod and Sham groups (both *p* < 0.01, Figure 2). There was no statistically significant difference in tear volume between the EA and Flu groups on day 35 (*p* > 0.05). The above results demonstrate that EA treatment promotes tear secretion and increases tear flow in DED mice.

### 3.2 EA treatment reduces corneal epithelial damage in DED mice

**Figure 3:**
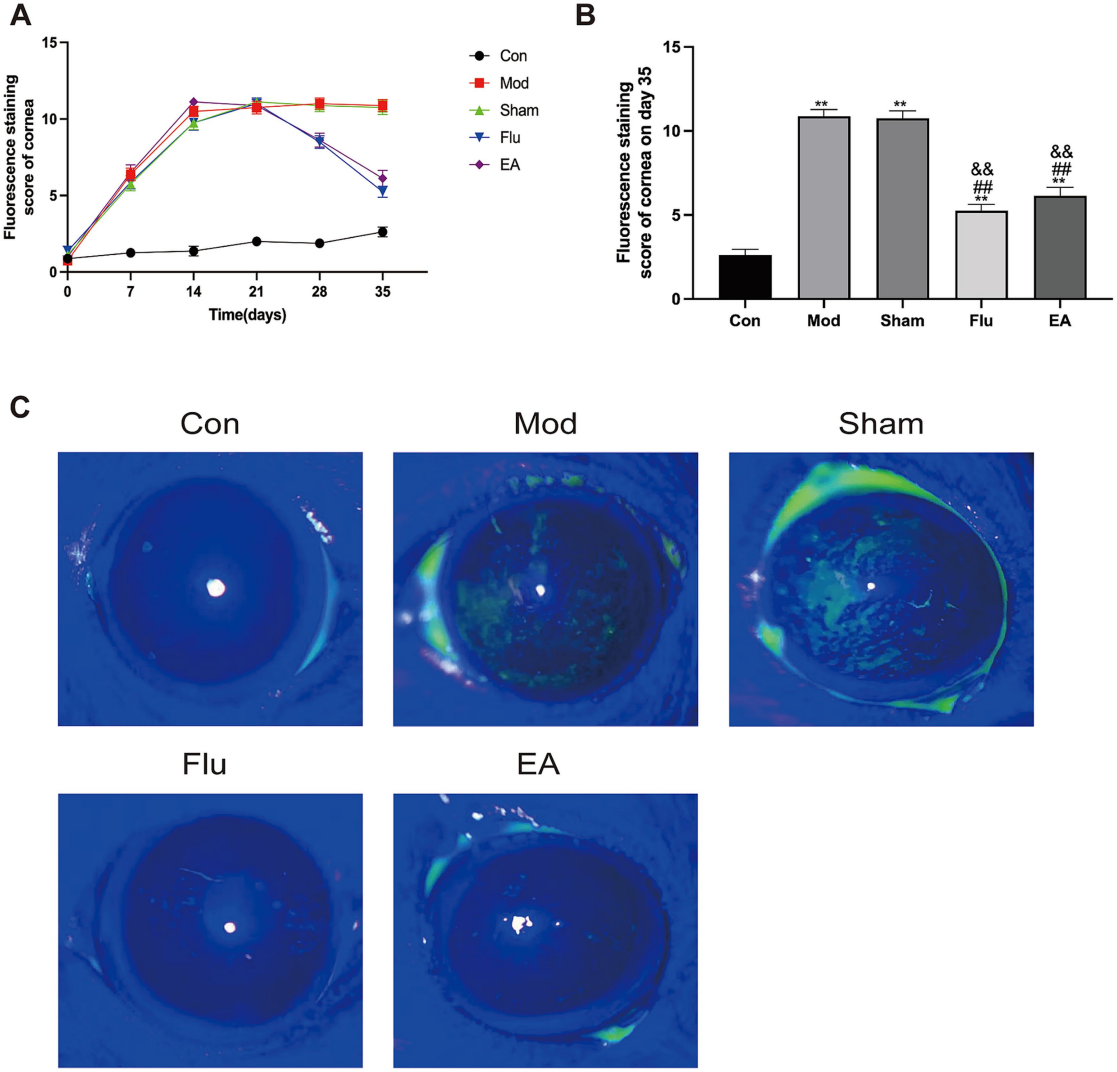
Effect of EA on corneal epithelial damage in scopolamine-hydrobromide-induced DED mice. **(A)** FL score changes at different time points (*n* = 8). **(B)** FL score of each group on the 35th day (*n* = 8). **(C)** Fluorescent staining pictures of the cornea. Compared with the Con group, ^∗∗^*p* < 0.01; compared with the Mod group, ^##^*p* < 0.01; compared with the Sham group, ^&&^*p* < 0.01.

FL allows the most visualization of the extent of corneal epithelial damage in DED. The FL scores in this study showed a statistically significant increase in the Mod, Sham, Flu and EA groups on day 21 compared to the same group before the experiment (all *p* < 0.01), and the scores of the Mod group on days 28 and 35 were statistically higher than in the pre-experimental period (both *p* < 0.01), proving that the modeling was effective (Figure 3A). The FL score was significantly lower in the EA group compared to the Mod and Sham groups on day 35 (6.13 ± 1.46 vs. 10.88 ± 1.13 points, 6.13 ± 1.46 vs. 10.75 ± 1.28 points, *p* < 0.01, Figure 3B). In addition, the FL was significantly lower in the Flu group than in the Mod and sham groups (both *p* < 0.01, Figure 3B). The FL scores of the EA and Flu groups on day 35 showed no significant difference (*p* > 0.05). Figure 3C shows the corneal fluorescence staining on day 35. The corneal epithelium of mice in the Con group was barely stained, while the corneal epithelium of mice in the Mod and Sham groups had marked staining. The staining of the ocular surface in the EA and Flu groups was reduced after the treatment, and the staining was scattered in spots.

### 3.3 EA treatment inhibits corneal stromal inflammation in DED mice

**Figure 4:**
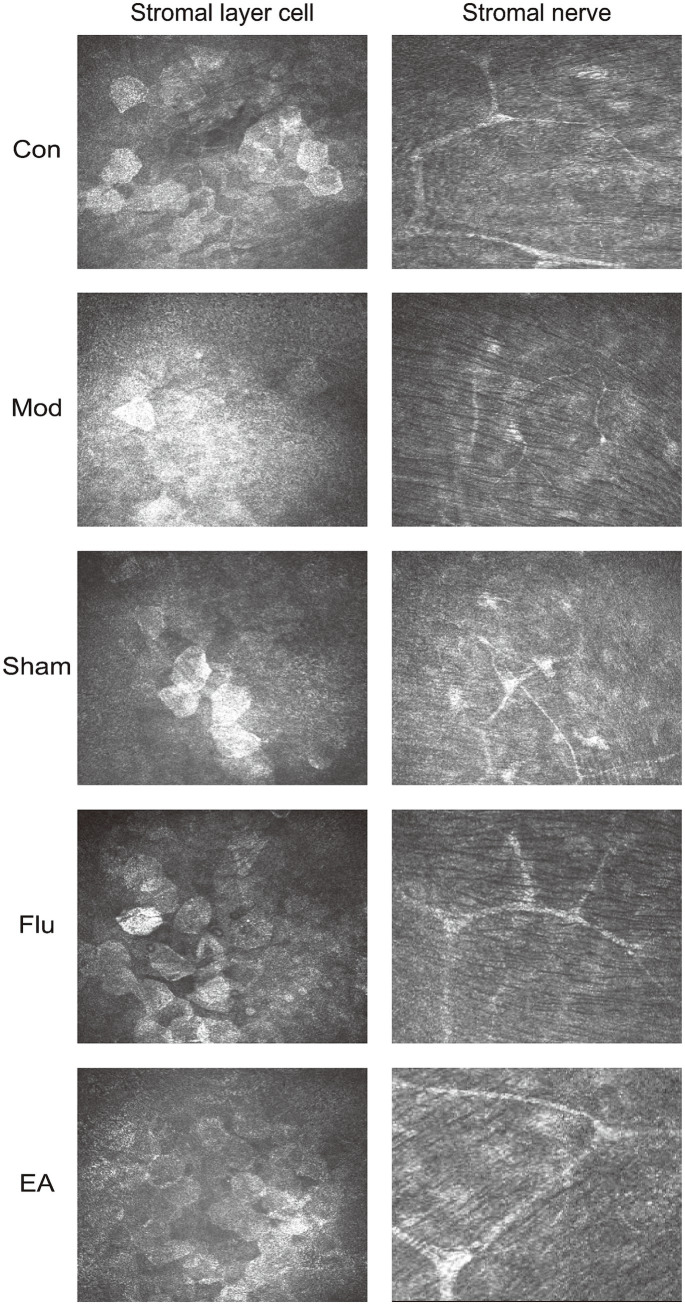
Effect of EA on corneal stromal inflammation in scopolamine-hydrobromide-induced DED mice. The IVCM images of the anterior and posterior stromal layers under the cornea on the 35th day (x800).

The corneal epithelium was observed under the corneal confocal microscopy, which is difficult to see using C57BL/6J mouse as an animal model (Figure 4). The Con group showed normal levels of stromal cells and nerve fiber distribution. The Mod group and the Sham group showed irregularly sized activated stromal cells, and intermittently thinner nerve fibers with a high degree of tortuosity. Compared with these two groups, the EA and Flu groups showed that the morphology and size of stromal cells gradually returned to normal except mild activation of partial cells, and there were no obvious abnormalities in the distribution of nerve fibers which indicate a significant reduction in corneal stromal inflammation.

### 3.4 EA treatment improves the morphological structure of the cornea and lacrimal gland in DED mice

**Figure 5:**
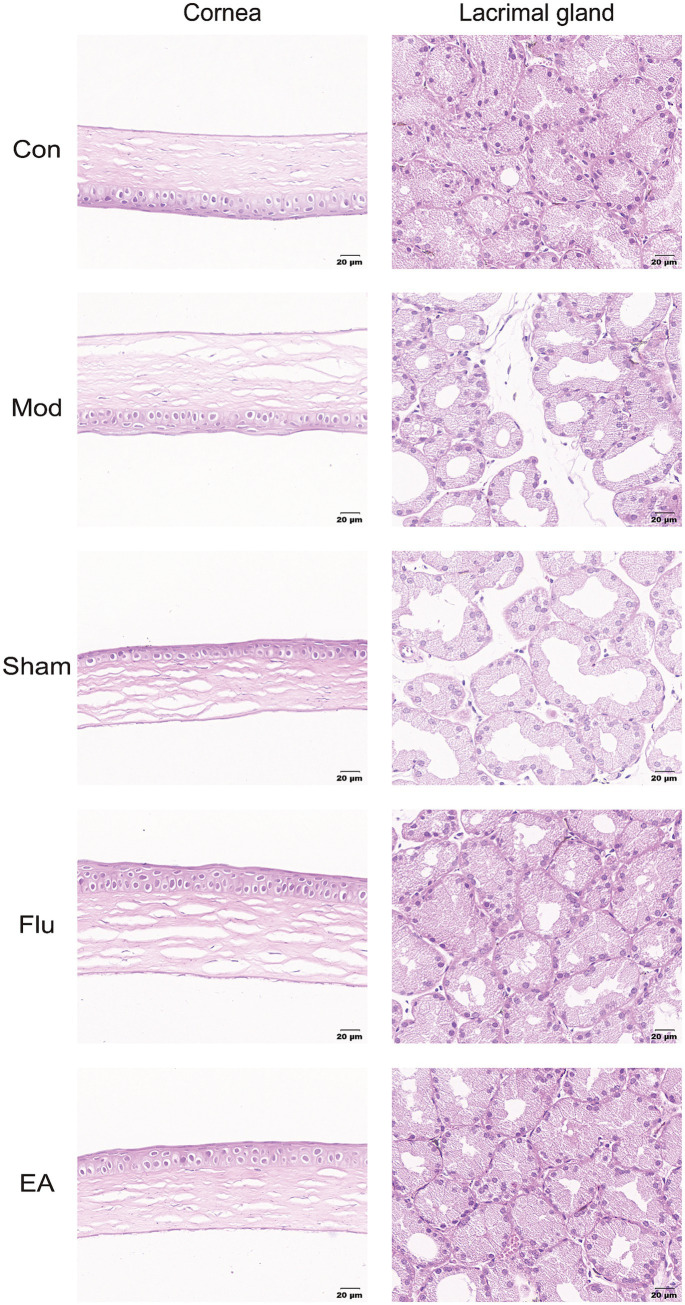
Effect of EA on morphological structure of the cornea and lacrimal gland in scopolamine-hydrobromide-induced DED mice. HE Staining pictures of the cornea and lacrimal gland.

The results of HE staining showed that the corneal tissue surface of the Mod and Sham groups were found to have reduced corneal cell layers, detached surface epithelial cells, disordered arrangement and swelling of collagen fibers in the matrix layer, and swelling. Additionally, obvious atrophy of lacrimal gland epithelial cells, expanded glandular cavity, and focal lymphocyte infiltration were also seen in the Mod and Sham groups. In comparison to these two groups, the EA and Flu groups had basically normal numbers of corneal epithelium layers, no apparent epithelium cell sloughing, well-arranged collagen fibers in the stromal layer, and slight swelling. In addition, the epithelial cells of the lacrimal gland tissue in the EA and Flu groups were partially atrophied, and the lumen of the gland was partially dilated, with a small amount of lymphocyte infiltration (Figure 5).

### 3.5 EA treatment modulates HMGB1 expression to inhibit ocular surface inflammation in DED mice

**Figure 6:**
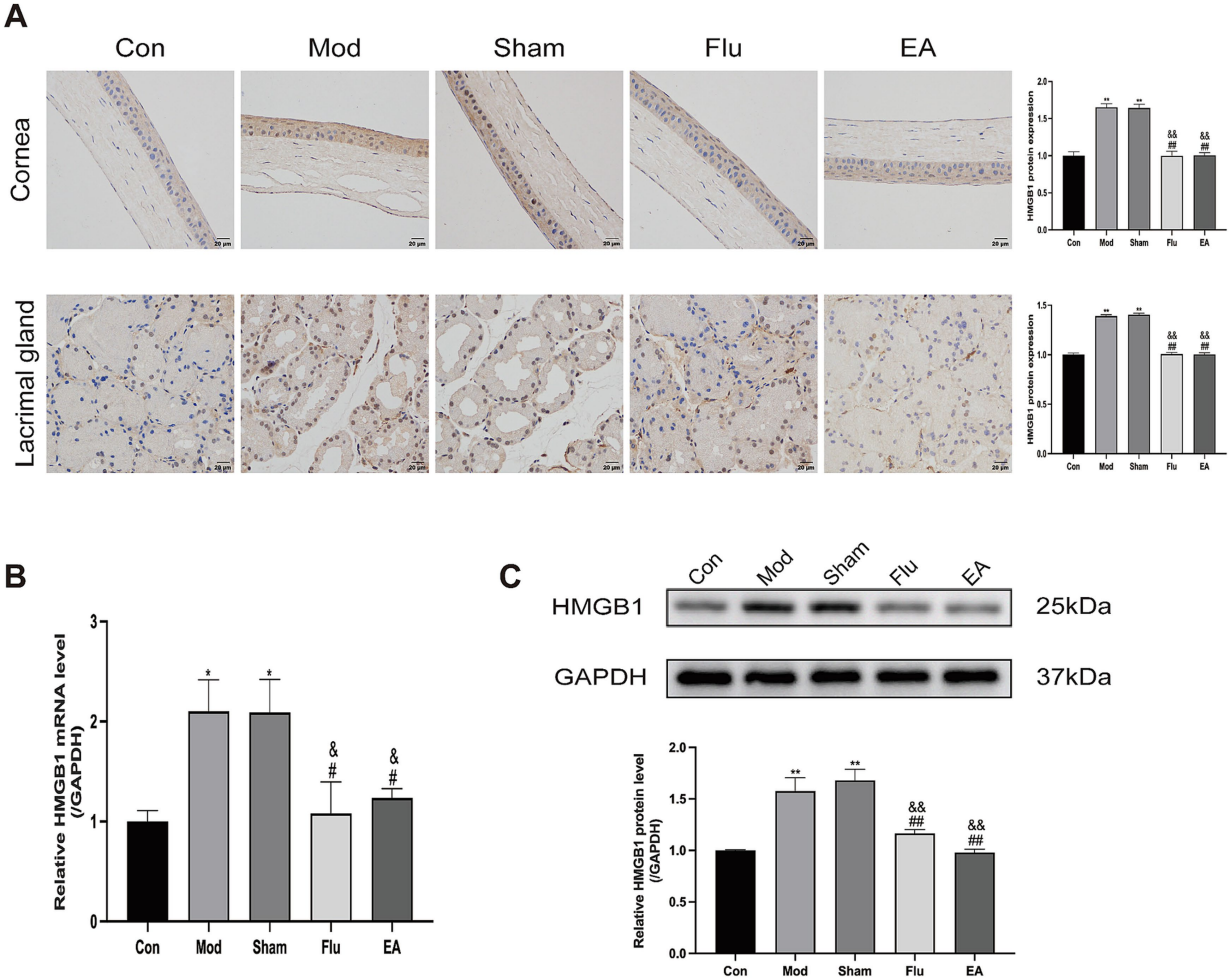
Effect of EA on expression of *HMGB1* in corneal and lacrimal gland tissues of scopolamine-hydrobromide-induced DED mice. **(A)** Immunohistochemical detection of HMGB1 expression in corneal and lacrimal gland tissues of DED mice (*n* = 3). **(B)** RT-qPCR detection of *HMGB1 mRNA* expression in lacrimal gland tissues of DED mice (*n* = 3). **(C)** WB detection of HMGB1 protein expression in lacrimal gland tissues of DED mice (*n* = 3). Compared with the Con group, ^*^p < 0.05, ^∗∗^*p* < 0.01; compared with the Mod group, ^#^*p* < 0.05, ^##^*p* < 0.01; compared with the Sham group, ^&^*p* < 0.05, ^&&^*p* < 0.01.

Our study analyzed the expression level of *HMGB1* in DED Mice to investigate whether EA treatment can inhibit dry eye surface inflammation by regulating the expression of *HMGB1*. Protein levels of HMGB1 in corneal and lacrimal gland tissues were detected by immunohistochemistry, and *mRNA* levels of *HMGB1* in lacrimal gland tissues were detected by real-time quantitative polymerase chain reaction (RT-qPCR) (Figures 6A,B). We found that *HMGB1* expression levels were significantly increased in the Mod and Sham groups, compared with the Con group (both *p* < 0.05). Meanwhile, the expression levels of *HMGB1* were significantly downregulated in the EA and Flu groups compared with the Mod and Sham groups (all *p* < 0.05) while there was no significant difference in HMGB1 expression levels between the EA and Flu groups (*p* > 0.05). Additionally, the protein expression levels of HMGB1 in lacrimal gland tissues were analyzed by WB (Figure 6C). The protein expression levels of HMGB1were significantly higher in the Mod and Sham groups than in the Con group (both *p* < 0.01). Additionally, the protein expression levels of HMGB1in the EA and Flu groups were significantly reduced compared to those in the Mod and Sham groups (all *p* < 0.01), but no significant difference in HMGB1 expression levels was observed between the EA and Flu groups (*p* > 0.05).

### 3.6 EA treatment inhibits the *HMGB1*-related signaling pathways in dry eye mice

**Figure 7:**
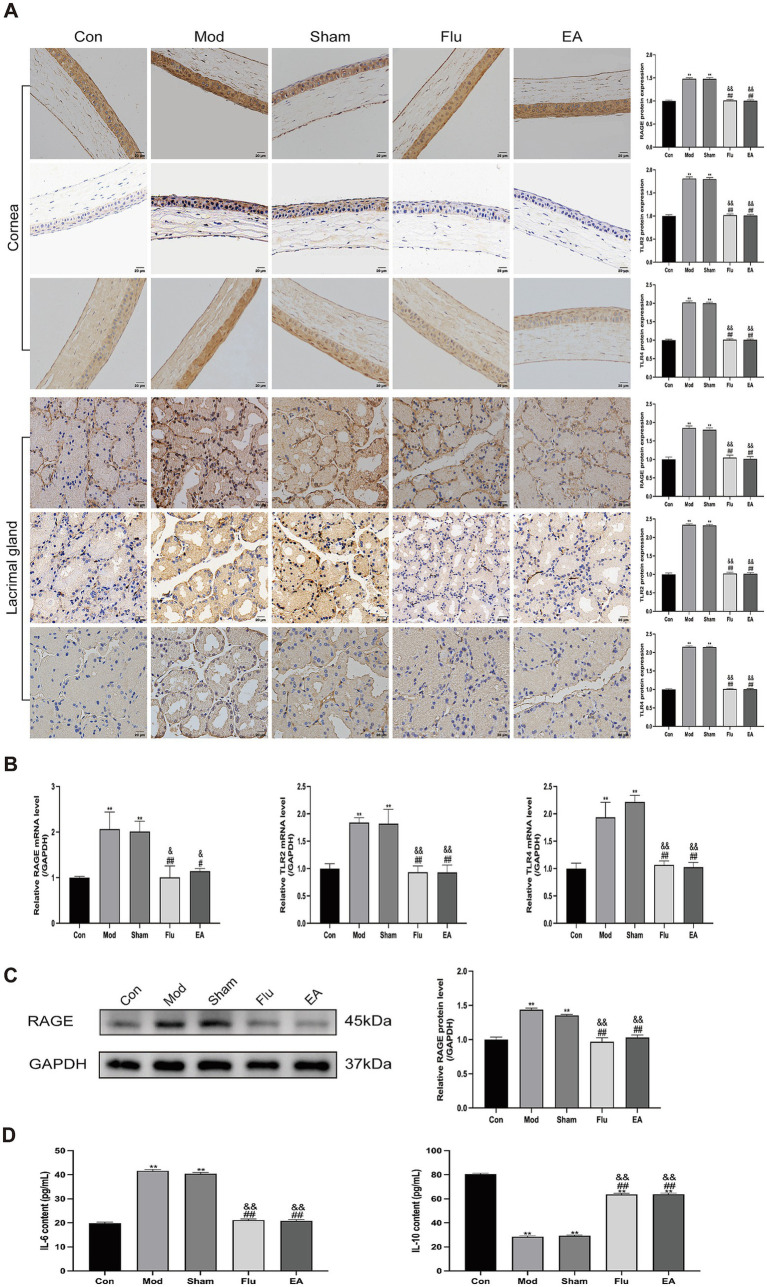
Effect of EA on expression of *RAGE*, *TLR2*, and *TLR4* in corneal and lacrimal gland tissues of scopolamine-hydrobromide-induced DED mice. **(A)** Immunohistochemical detection of RAGE, TLR2, and TLR4 expression in corneal and lacrimal gland tissues of DED mice (*n* = 3). **(B)** RT-qPCR detection of *RAGE*, *TLR2*, and *TLR4 mRNA* expression in lacrimal gland tissues of DED mice (*n* = 3). **(C)** WB detection of RAGE protein expression in lacrimal gland tissues of DED mice (*n* = 3). **(D)** ELISA detection of IL-6 and IL-10 levels in serum of DED mice (*n* = 6). Compared with the Con group, ^∗∗^*p* < 0.01; compared with the Mod group, ^#^*p* < 0.05, ^##^*p* < 0.01; compared with the Sham group, ^&^*p* < 0.05, ^&&^*p* < 0.01.

In this study, we determined whether the anti-inflammatory effect of EA treatment on ocular surface inflammation in DED mice was associated with the *HMGB1*-related signaling pathways by further detecting the expression of *HMGB1* receptors and downstream related factors. Protein levels of *HMGB1* receptors and downstream related factors in corneal and lacrimal gland tissues were detected by immunohistochemistry, and *mRNA* levels of these indicators in lacrimal gland tissues were detected by RT-qPCR (Figures 7A,B). We found that *RAGE* (both *p* < 0.01), *TLR2* (both *p* < 0.01), and *TLR4* (both *p* < 0.01) expression levels were significantly increased in the Mod and Sham groups, compared with the Con group. Meanwhile, the expression levels of *RAGE* (all *p* < 0.05), *TLR2* (all *p* < 0.01), and *TLR4* (all *p* < 0.01) were significantly downregulated in the EA and Flu groups compared with the Mod and Sham groups. However, there was no significant difference in the expression levels of these indicators between the EA and Flu groups (all *p* > 0.05). Additionally, the protein expression levels of RAGE in lacrimal gland tissues were analyzed by WB (Figure 7C). The protein expression levels of RAGE were significantly higher in the Mod and Sham groups than in the Con group (both *p* < 0.01), and the protein expression levels of RAGE in the EA and Flu groups were significantly reduced compared to those in the Mod and Sham groups (all *p* < 0.01). There was no statistically significant difference in the protein expression levels of RAGE between the EA and Flu groups (*p* > 0.05). Meanwhile, the ELISA results in the serum showed that the levels of IL-6 (both *p* < 0.01) were significantly increased, and the levels of IL-10 (both *p* < 0.01) were significantly decreased in the Mod and Sham groups, compared with the Con group (Figure 7D). Compared with the Mod and Sham groups, the levels of IL-6 (all *p* < 0.01) were significantly downregulated and the levels of IL-10 (all *p* < 0.01) were significantly increased in the EA and Flu groups (Figure 7D). However, there was no significant difference in the levels of IL-6 and IL-10 between the EA and Flu groups (all *p* > 0.05).

These results showed that the trend of EA decreasing the expression of *HMGB1* and its receptors was consistent in both the protein and transcriptional levels, and EA could downregulate the level of pro-inflammatory cytokine IL-6 and promote the production of anti-inflammatory cytokine IL-10, indicating that EA may inhibit ocular surface inflammation in DED mice by suppressing the *HMGB1*-related signaling pathways.

## 4 Discussion

DED is characterized by an imbalance of tear film homeostasis, which can be accompanied by ocular surface inflammation, tissue damage, and neurosensory abnormalities (31). The main clinical manifestations of DED are dry eyes, eye distension, photophobia, eye pain, and fluctuating visual acuity. In severe cases, DED can lead to irreversible visual impairment due to corneal epithelial defects, ulcers, and even scar formation (32). Although the pathogenesis of DED remains incompletely understood, inflammation, resulting from early innate immune and adaptive responses, has been identified as a key factor that may trigger a DED vicious cycle (33, 34). Related studies have indicated that EA has anti-inflammatory effects, but its mechanism of action is not completely understood (17, 18, 35). EA treatment is the process of inserting needles into specific acupoints and then combining the needles with electrical stimulation, causing local muscles to continuously beat at a certain frequency. This sustained physical stimulation can activate cell activity and promote the regeneration of cells and glands. Today, EA is widely used in the treatment of various ophthalmic diseases, including myopia, ophthalmoplegia, glaucoma, and nystagmus (36, 37). EA has been recognized and applied by many countries due to its effectiveness and safety. According to numerous clinical reports, EA can be used to treat DED and improve patient midterm outcomes such as DED symptoms, tear secretion and tear film stability (11–13). Consequently, exploring therapeutic mechanism of EA on DED is of great significance. In this study, the evaluation was done using a scopolamine hydrobromide induced mouse model of DED. Our study found that the DED mice model exhibited reduced tear flow, corneal and lacrimal gland epithelial damage, corneal stromal cell activation, and abnormal corneal nerve morphology, indicating successful model preparation, which has strong similarities to the inflammation and damage arouse from human DED (38). We provided new evidence for the use of EA in the treatment of DED in the present study by showing that EA could alleviate the degree of damage to corneal and lacrimal gland tissues in DED mice.

*HMGB1* was initially recognized as a highly conserved *DNA*-binding protein in the nucleus, and across multiple species, involved in gene transcription, maintenance of nucleosome structure, and tissue degeneration (39). *HMGB1* is an important late-stage inflammatory factor that is actively secreted by monocytes or macrophages stimulated by inflammatory factors or passively released from necrotic cells. It can also serve as a molecule for extracellular DAMPs, promoting inflammation, cell differentiation, and cell migration (40). *HMGB1* released into the extracellular space mediates the activation of innate immune responses. It binds differentially to the receptor, which in turn induces inflammatory and immune responses in the body and exacerbates tissue damage (41, 42). In almost all types of inflammation, research has found that *HMGB1* is overexpressed, and targeting *HMGB1* represents a new target for inflammation therapy (43–45). Experimental results have shown that inhibiting *HMGB1* can significantly reduce the production of pro-inflammatory cytokines interleukin-1β (*IL-1β*), interleukin-6 (*IL-6*) and interleukin-8 (*IL-8*) (46). Our study detected the expression of *HMGB1*, and the results showed that the expression trends of *HMGB1* in the cornea of the Mod and Sham groups were the same, and both of them were significantly higher than that of the Con group. Similarly, the expression results of *HMGB1* detected in lacrimal gland tissues were consistent with those detected in corneal tissues, indicating that the DED model activated the expression of *HMGB1*. It was found that EA significantly reduced the expression of *HMGB1*, similar to the effect of fluorometholone. The above results indicated that EA treatment for DED may be achieved by the downregulation of HMGB1.

Current research indicates that *HMGB1* plays an important role in activating ocular surface inflammatory factors, and it can stimulate downstream signaling pathways, which is key to dry eye formation (47). *RAGE*, one of the earliest discovered *HMGB1* receptors, is the primary pathway for immune signaling in inflammatory diseases. It has been demonstrated that corneal inflammation activates the expression of *HMGB1* and *RAGE* (48). The combination of *HMGB1* and *RAGE* can activate the phosphatidylinositol 3-kinase/protein kinase B (PI3K/AKT) and mitogen-activated protein kinase (MAPK) pathways, leading to the activation of nuclear factor kappa-B (*NF-κB*) (49). Furthermore, *TLRs* are also the main receptors involved in DAMP recognition, especially *TLR2* and *TLR4* (50, 51). *HMGB1* can also activate the *NF-κB* inflammatory pathway by binding to *TLRs*, leading to an inflammatory response (40, 52, 53). IL-6, as a typical pro-inflammatory cytokine, is a classic target gene of *NF-κB*, participating in initiating inflammatory signals, while *IL-10* is a key anti-inflammatory cytokine. When *NF-κB* is strongly activated, it consumes a large amount of limited transcription coactivators in the cell to drive the transcription of pro-inflammatory genes such as *IL-6* and *TNF-α*, resulting in a severe shortage of coactivators available for *IL-10* gene transcription, indirectly inhibiting the expression of *IL-10* (54, 55). Previous studies have shown that *HMGB1* antagonists effectively reduce mortality in a mouse sepsis model by blocking the *HMGB1*-*RAGE* signaling pathway (56, 57). Also, studies have found that the *HMGB1*-*TLR2* signaling pathway promotes neutrophil programmed death ligand 1 (*PD-L1*) expression and mediates T-lymphocyte apoptosis, leading to immunosuppression in sepsis (58). Meanwhile, the *HMGB1*-*TLR4* pathway has been found to play an important role in the neurotoxic inflammatory response (59, 60). Thus, *HMGB1* initiates complex signaling pathways such as *NF-κB* pathways by binding to *RAGE*, *TLR2*, or *TLR4*, which can promote the production of pro-inflammatory cytokines and inhibit the production of anti-inflammatory cytokines, leading to the occurrence and development of ocular surface inflammation. However, whether EA inhibits DED surface inflammation by modulating the *HMGB1*-related signaling pathways is still unclear. Our study investigated the effects of EA on *HMGB1*-related signaling pathways by detecting the expression of *RAGE*, *TLR2*, *TLR4*, IL-6, and IL-10 in DED mice. The results showed that the expression of *RAGE*, *TLR2*, and *TLR4* was significantly higher in the Mod and Sham groups than in the Con group, both in the corneal and lacrimal tissues. But the expression of *RAGE*, *TLR2*, and *TLR4* was significantly reduced after EA treatment, which was similar to the effect of fluorometholone. Meanwhile, the level of pro-inflammatory cytokine IL-6 decreased, and the level of anti-inflammatory cytokine IL-10 increased after EA treatment. Therefore, we suggest that EA may inhibit ocular surface inflammation in DED mice by modulating the *HMGB1*-related signaling pathways. Overall, it can be seen that EA has a clear therapeutic effect on inhibiting ocular surface inflammation in DED mice, and its mechanism may be related to the regulation of *HMGB1*-related signaling pathways by EA, providing a basis for the beneficial role of EA in the pathogenesis of DED. Although this study shows that the inhibition of ocular surface inflammation in DED by EA may be related to the *HMGB1*-related signaling pathways, it has not been further verified. We intend to use pathway agonists to further explore *HMGB1*-related signaling pathways in future studies.

## 5 Conclusion

We investigated the effects of EA on DED mice induced by scopolamine hydrobromide and the underlying mechanisms of this process. Our results suggest that EA may inhibit ocular surface inflammation in DED mice by regulating the *HMGB1*-related signaling pathways. This study provides a theoretical basis for anti-inflammatory mechanism of EA in DED, and also offers a novel potential therapeutic target for treating DED.

## Data Availability

The raw data supporting the conclusions of this article will be made available by the authors, without undue reservation.

## References

[1] Craig JP Nichols KK Akpek EK Caffery B Dua HS Joo C-K. TFOS DEWS II definition and classification report. Ocul Surf. (2017) 15:276–83. doi: 10.1016/j.jtos.2017.05.008, PMID: 28736335

[2] Stapleton F Alves M Bunya VY Jalbert I Lekhanont K Malet F. TFOS DEWS II epidemiology report. Ocul Surf. (2017) 15:334–65. doi: 10.1016/j.jtos.2017.05.003, PMID: 28736337

[3] Stapleton F Argüeso P Asbell P Azar D Bosworth C Chen W. TFOS DEWS III Digest report. Am J Ophthalmol. (2025) 279:451–553. doi: 10.1016/j.ajo.2025.05.040 40472874

[4] McDonald M Patel DA Keith MS Snedecor SJ. Economic and humanistic burden of dry eye disease in Europe, North America, and Asia: a systematic literature review. Ocul Surf. (2016) 14:144–67. doi: 10.1016/j.jtos.2015.11.002, PMID: 26733111

[5] Wolffsohn JS Benítez-Del-Castillo J Loya-Garcia D Inomata T Iyar G Liang L. Tfos dews III diagnostic methodology. Am J Ophthalmol. (2025) 279:387–450. doi: 10.1016/j.ajo.2025.05.033 40451408

[6] Deswal J Arya SK Raj A Bhatti A. A case of bilateral corneal perforation in a patient with severe dry eye. J Clin Diagn Res. (2017) 11:ND01–2. doi: 10.7860/JCDR/2017/24149.9645, PMID: 28571178 PMC5449824

[7] Bron AJ de Paiva CS Chauhan SK Bonini S Gabison EE Jain S. TFOS DEWS II pathophysiology report. Ocul Surf. (2017) 15:438–510. doi: 10.1016/j.jtos.2017.05.011, PMID: 28736340

[8] Zhou J Sun Y Cheng Y Chen T Chen L. Efficacy of moisture chamber goggles combined with fluorometholone eye drops on visual function and oxidative stress in patients with dry eye disease. Am J Transl Res. (2024) 16:7782–91. doi: 10.62347/puny8378, PMID: 39822521 PMC11733328

[9] Jones L Downie LE Korb D Benitez-del-Castillo JM Dana R Deng SX. TFOS DEWS II management and therapy report. Ocul Surf. (2017) 15:575–628. doi: 10.1016/j.jtos.2017.05.006, PMID: 28736343

[10] Longhurst JC. Defining meridians: a modern basis of understanding. J Acupunct Meridian Stud. (2010) 3:67–74. doi: 10.1016/S2005-2901(10)60014-3, PMID: 20633518

[11] Yang G Kong X Guo X Yang Y Xie C Lu Y. Effects of electroacupuncture on dry eye: a pilot randomized controlled trial. Acta Ophthalmol. (2023) 101:e315–26. doi: 10.1111/aos.15271, PMID: 36245315

[12] Lu Y-Q Yang G Li M-Y Hong J Yang Y-T Wang X-J. Electroacupuncture for mild-to-moderate dry eye: study protocol for a multicentre, randomised, single-blind, sham-controlled trial. BMJ Open. (2023) 13:e069369. doi: 10.1136/bmjopen-2022-069369, PMID: 38056935 PMC10711924

[13] Wu M Tang Q Gao W Zhu L. A novel approach to the immediate effects of electroacupuncture on dry eye: a case series. Explore. (2024) 20:430–3. doi: 10.1016/j.explore.2023.10.010, PMID: 37891043

[14] Wang T-N Zhao J-Y Yang Y-C Zhou Z-X Feng Y-H Chen J-T. Acupuncture and moxibustion treatment for dry eye disease: a network meta-analysis of rando-mized controlled trial. Zhen Ci Yan Jiu. (2021) 46:1057–66. doi: 10.13702/j.1000-0607.20210292, PMID: 34970884

[15] Sun XY Shen HX Liu CY Zheng XJ Zhao Y Zhou JB. Acupuncture mitigates ocular surface inflammatory response via α7nAChR/NF-κB p65 signaling in dry eye guinea pigs. Zhen Ci Yan Jiu. (2022) 47:975–82. doi: 10.13702/j.1000-0607.20220015, PMID: 36453674

[16] Ding N Wei Q Xu Q Liu C Ni Y Zhao J. Acupuncture alleviates corneal inflammation in New Zealand white rabbits with dry eye diseases by regulating α7nAChR and NF-κB signaling pathway. Evid Based Complement Alternat Med. (2022) 2022:1–12. doi: 10.1155/2022/6613144, PMID: 36419616 PMC9678486

[17] Wan M-M Fu Z-Y Jin T Wang Z-Y Sun X-Y Gao W-P. Electroacupuncture regulates the P2X7R-NLRP3 inflammatory cascade to relieve decreased sensation on ocular surface of type 2 diabetic rats with dry eye. Purinergic Signal. (2024) 21:651–66. doi: 10.1007/s11302-024-09991-0, PMID: 38467962 PMC12454788

[18] Ding N Wei Q Deng W Sun X Zhang J Gao W. Electroacupuncture alleviates inflammation of dry eye diseases by regulating the *α* 7nAChR/NF- *κ* b signaling pathway. Oxidative Med Cell Longev. (2021) 2021:6673610. doi: 10.1155/2021/6673610, PMID: 33897942 PMC8052151

[19] Zhang D Zhao Y Yang Y-T Zhao Y Wu D-Y Liu X-X. A mechanism study of Electroacupuncture for dry eye syndrome by targeting conjunctival cytokine expressions. Curr Eye Res. (2020) 45:419–27. doi: 10.1080/02713683.2019.1666997, PMID: 31557061

[20] ChenRZouJZhongXLiuJKangRTangD. DAMP signaling networks: from receptors to diverse pathophysiological functions. J Adv Res. (2025). 31:S2090-1232(25)00577-6. doi: 10.1016/j.jare.2025.07.047, PMID: 40752681

[21] Ma M Jiang W Zhou R. DAMPs and DAMP-sensing receptors in inflammation and diseases. Immunity. (2024) 57:752–71. doi: 10.1016/j.immuni.2024.03.002, PMID: 38599169

[22] Xu X Piao HN Aosai F Zeng XY Cheng JH Cui YX. Arctigenin protects against depression by inhibiting microglial activation and neuroinflammation via HMGB1/TLR4/NF-κB and TNF-α/TNFR1/NF-κB pathways. Br J Pharmacol. (2020) 177:5224–45. doi: 10.1111/bph.15261, PMID: 32964428 PMC7589024

[23] Wang J Li R Peng Z Hu B Rao X Li J. HMGB1 participates in LPS-induced acute lung injury by activating the AIM2 inflammasome in macrophages and inducing polarization of M1 macrophages via TLR2, TLR4, and RAGE/NF-κB signaling pathways. Int J Mol Med. (2020) 45:61–80. doi: 10.3892/ijmm.2019.4402, PMID: 31746367 PMC6889921

[24] Li J Wang FP She WM Yang CQ Li L Tu CT. Enhanced high-mobility group box 1 (HMGB1) modulates regulatory T cells (Treg)/T helper 17 (Th17) balance via toll-like receptor (TLR)-4-interleukin (IL)-6 pathway in patients with chronic hepatitis B. J Viral Hepat. (2014) 21:129–40. doi: 10.1111/jvh.12152, PMID: 24383926

[25] Zhang J Chen L Wang F Zou Y Li J Luo J. Extracellular HMGB1 exacerbates autoimmune progression and recurrence of type 1 diabetes by impairing regulatory T cell stability. Diabetologia. (2020) 63:987–1001. doi: 10.1007/s00125-020-05105-8, PMID: 32072192 PMC7145789

[26] Ge Y Huang M Yao Y-m. The effect and regulatory mechanism of high mobility group Box-1 protein on immune cells in inflammatory diseases. Cells. (2021) 10:1044. doi: 10.3390/cells10051044, PMID: 33925132 PMC8145631

[27] Zhang S Hu L Jiang J Li H Wu Q Ooi K. HMGB1/RAGE axis mediates stress-induced RVLM neuroinflammation in mice via impairing mitophagy flux in microglia. J Neuroinflammation. (2020) 17:15. doi: 10.1186/s12974-019-1673-3, PMID: 31924219 PMC6953162

[28] Hakim FE Farooq AV. Dry eye disease: an update in 2022. JAMA. (2022) 327:478–9. doi: 10.1001/jama.2021.19963, PMID: 35103781

[29] Cheng J Pan G Wang Z Chu H Pu Y. Electroacupuncture treatment enhances synaptic plasticity in middle cerebral artery occlusion mice via the miR-670-3p/HMGB1 axis. J Chin Med Assoc. (2025) 88:520–9. doi: 10.1097/jcma.0000000000001226, PMID: 40090958 PMC12637104

[30] Liang Q Guo R Tsao JR He Y Wang C Jiang J. Salidroside alleviates oxidative stress in dry eye disease by activating autophagy through AMPK-Sirt1 pathway. Int Immunopharmacol. (2023) 121:110397. doi: 10.1016/j.intimp.2023.110397, PMID: 37302369

[31] O'Neil EC Henderson M Massaro-Giordano M Bunya VY. Advances in dry eye disease treatment. Curr Opin Ophthalmol. (2019) 30:166–78. doi: 10.1097/icu.0000000000000569, PMID: 30883442 PMC6986373

[32] Yokoi N Georgiev GA. Tear-film-oriented diagnosis for dry eye. Jpn J Ophthalmol. (2019) 63:127–36. doi: 10.1007/s10384-018-00645-4, PMID: 30783943

[33] Rhee MK Mah FS. Inflammation in dry eye disease: how do we break the cycle? Ophthalmology. (2017) 124:S14–9. doi: 10.1016/j.ophtha.2017.08.029, PMID: 29055357

[34] Li S Lu Z Huang Y Wang Y Jin Q Shentu X. Anti-oxidative and anti-inflammatory micelles: break the dry eye vicious cycle. Adv Sci. (2022) 9:e2200435. doi: 10.1002/advs.202200435, PMID: 35435328 PMC9189644

[35] Fu Z Wan M Jin T Lai S Li X Sun X. Electroacupuncture modulates the TLR4-NF-κB inflammatory signaling pathwayto attenuate ocular surface inflammation in dry eyes of type 2 diabetic rats. Cell Mol Biol. (2024) 70:111–8. doi: 10.14715/cmb/2024.70.5.15 38814228

[36] Liu Y Hao Q Lu X Wang P Guo D Zhang X. Electroacupuncture improves retinal function in myopia Guinea pigs probably via inhibition of the RhoA/ROCK2 signaling pathway. Heliyon. (2024) 10:e35750. doi: 10.1016/j.heliyon.2024.e35750, PMID: 39170407 PMC11337061

[37] Huang Y Hu H He K Li X Chen Q Ma R. Acupuncture for abducens nerve palsy after radiochemotherapy: a CARE-compliant case report. Explore. (2023) 19:469–74. doi: 10.1016/j.explore.2022.06.005, PMID: 35715324

[38] Dursun D Wang M Monroy D Li D-Q Lokeshwar BL Stern ME. A mouse model of keratoconjunctivitis sicca. Invest Ophthalmol Vis Sci. (2002) 43:632–8. 11867577

[39] Iba T Nakarai E Takayama T Nakajima K Sasaoka T Ohno Y. Combination effect of antithrombin and recombinant human soluble thrombomodulin in a lipopolysaccharide induced rat sepsis model. Crit Care. (2009) 13:R203. doi: 10.1186/cc8210, PMID: 20003418 PMC2811901

[40] Zhang Q-Y Wu L-Q Zhang T Han Y-F Lin X. Autophagy-mediated HMGB1 release promotes gastric cancer cell survival via RAGE activation of extracellular signal-regulated kinases 1/2. Oncol Rep. (2015) 33:1630–8. doi: 10.3892/or.2015.3782, PMID: 25652880 PMC4358082

[41] Andersson U Yang H Harris H. Extracellular HMGB1 as a therapeutic target in inflammatory diseases. Expert Opin Ther Targets. (2018) 22:263–77. doi: 10.1080/14728222.2018.1439924, PMID: 29447008

[42] Kim SY Son M Lee SE Park IH Kwak MS Han M. High-mobility group box 1-induced complement activation causes sterile inflammation. Front Immunol. (2018) 9:705. doi: 10.3389/fimmu.2018.00705, PMID: 29696019 PMC5904255

[43] Wang S Zhang Y. HMGB1 in inflammation and cancer. J Hematol Oncol. (2020) 13:116. doi: 10.1186/s13045-020-00950-x 32831115 PMC7443612

[44] Fu Y Xiang Y Wang Y Liu Z Yang D Zha J. The STAT1/HMGB1/NF-κB pathway in chronic inflammation and kidney injury after cisplatin exposure. Theranostics. (2023) 13:2757–73. doi: 10.7150/thno.81406, PMID: 37284446 PMC10240827

[45] Hu M Zhang Y Lu Y Han J Guo T Cui P. Regulatory mechanisms of HMGB1 and its receptors in polycystic ovary syndrome-driven gravid uterine inflammation. FEBS J. (2023) 290:1874–906. doi: 10.1111/febs.16678, PMID: 36380688 PMC10952262

[46] Sun H Chu X Qian W Yin H. CircMYO1C silencing alleviates chondrocytes inflammation and apoptosis through m(6)a/HMGB1 axis in osteoarthritis. Biotechnol Appl Biochem. (2024) 71:1360–9. doi: 10.1002/bab.2635, PMID: 38982736

[47] Park HS Kim EN Kim MY Lim JH Kim HW Park CW. The protective effect of neutralizing high-mobility group box1 against chronic cyclosporine nephrotoxicity in mice. Transpl Immunol. (2016) 34:42–9. doi: 10.1016/j.trim.2015.11.001, PMID: 26603313

[48] Miyazaki D Kandori-Inoue M Shimizu Y Ohtani F Chono I Inoue Y. Role played by receptors for advanced glycosylation end products in corneal endothelial cells after HSV-1 infection. Int J Mol Sci. (2021) 22:5833. doi: 10.3390/ijms22115833, PMID: 34072468 PMC8199122

[49] Liu C Zhang H Guan R Zou Y Chen M Du M. Inhibition of the HMGB1-RAGE Axis attenuates microglial inflammation and ameliorates hypoxia-induced cognitive impairment. Int J Mol Sci. (2025) 26:8782. doi: 10.3390/ijms26188782, PMID: 41009351 PMC12469424

[50] De Luca G Goette NP Lev PR Baroni Pietto MC Marin Oyarzún CP Castro Ríos MA. Elevated levels of damage-associated molecular patterns HMGB1 and S100A8/A9 coupled with toll-like receptor-triggered monocyte activation are associated with inflammation in patients with myelofibrosis. Front Immunol. (2024) 15:1365015. doi: 10.3389/fimmu.2024.1365015, PMID: 39391311 PMC11465240

[51] Crews FT Coleman LG Jr Macht VA Vetreno RP. Alcohol, HMGB1, and innate immune signaling in the brain. Alcohol Res. (2024) 44:04. doi: 10.35946/arcr.v44.1.04, PMID: 39135668 PMC11318841

[52] Andersson U Yang H Harris H. High-mobility group box 1 protein (HMGB1) operates as an alarmin outside as well as inside cells. Semin Immunol. (2018) 38:40–8. doi: 10.1016/j.smim.2018.02.011, PMID: 29530410

[53] Bertheloot D Latz E. HMGB1, IL-1α, IL-33 and S100 proteins: dual-function alarmins. Cell Mol Immunol. (2017) 14:43–64. doi: 10.1038/cmi.2016.34, PMID: 27569562 PMC5214941

[54] Saraiva M O'Garra A. The regulation of IL-10 production by immune cells. Nat Rev Immunol. (2010) 10:170–81. doi: 10.1038/nri2711, PMID: 20154735

[55] La S Abaidullah M Li H Cui Y Liu B Shi Y. Alfalfa polysaccharide alleviates colitis by regulating intestinal microbiota and the intestinal barrier against the TLR4/MyD88/NF-κB pathway. Nutrients. (2025) 17:3001. doi: 10.3390/nu17183001, PMID: 41010526 PMC12472594

[56] Meng G Rutz M Schiemann M Metzger J Grabiec A Schwandner R. Antagonistic antibody prevents toll-like receptor 2-driven lethal shock-like syndromes. J Clin Invest. (2004) 113:1473–81. doi: 10.1172/jci20762, PMID: 15146245 PMC406529

[57] Gasparotto J Girardi CS Somensi N Ribeiro CT Moreira JCF Michels M. Receptor for advanced glycation end products mediates sepsis-triggered amyloid-β accumulation, tau phosphorylation, and cognitive impairment. J Biol Chem. (2018) 293:226–44. doi: 10.1074/jbc.M117.786756, PMID: 29127203 PMC5766916

[58] Liu J Song K Lin B Chen Z Zuo Z Fang Y. HMGB1 promotes neutrophil PD-L1 expression through TLR2 and mediates T cell apoptosis leading to immunosuppression in sepsis. Int Immunopharmacol. (2024) 133:112130. doi: 10.1016/j.intimp.2024.112130, PMID: 38648712

[59] Kleen JK Holmes GL. Taming TLR4 may ease seizures. Nat Med. (2010) 16:369–70. doi: 10.1038/nm0410-369, PMID: 20376038

[60] Maroso M Balosso S Ravizza T Liu J Aronica E Iyer AM. Toll-like receptor 4 and high-mobility group box-1 are involved in ictogenesis and can be targeted to reduce seizures. Nat Med. (2010) 16:413–9. doi: 10.1038/nm.2127, PMID: 20348922

